# Training US health care professionals on human trafficking: where do we go from here?

**DOI:** 10.1080/10872981.2017.1267980

**Published:** 2017-01-06

**Authors:** Clydette Powell, Kirsten Dickins, Hanni Stoklosa

**Affiliations:** ^a^ Department of Pediatrics, The George Washington University School of Medicine and Health Sciences, Washington, DC, USA; ^b^ College of Nursing, Rush University, Chicago, IL, USA; ^c^ Department of Emergency Medicine, Harvard Medical School, Brigham and Women’s Hospital, Boston, MA, USA

**Keywords:** Violencemedical education, community health, migrant health, adolescent health, emergency department, free clinics, social justice, sex trafficking, labor trafficking, patient–centered outcomes

## Abstract

Some 21 million adults and children are labor-trafficked or sex-trafficked through force, fraud, or coercion. In recognition of the interface between trafficking victims and the healthcare setting, over the last 10 years there has been a notable increase in training of health care professionals (HCPs) on human trafficking (HT) and its health implications. Many organizations have developed curricula and offered training in various clinical settings. However, methods and content of this education on trafficking vary widely, and there is little evaluation of the impact of the training. The goal of this study was to assess the gaps and strengths in HT education of HCPs in the US. This mixed-method study had two components. The first component consisted of structured interviews with experts in human trafficking HCP education. The second portion of the study involved an analysis of data from HCP calls to the National Human Trafficking Resource Center (NHTRC). The interviews captured trainer-specific data on types of HT training, duration and frequency, key content areas, presence of evaluation approaches and indicators, as well as an assessment of barriers and strengths in HT training for HCP. NHTRC call database analysis demonstrated increasing trends since 2008 in calls by HCPs. Overall findings revealed the need for standardization of HT training content to assure correct information, trauma-informed and patient-centered care, and consistent messaging for HCPs. Evaluation metrics for HT training need to be developed to demonstrate behavior change and impact on service delivery and patient-centered outcomes for HT victims, according to our proposed adapted Kirkpatrick’s Pyramid model. HT training and evaluation would benefit from an agency or institution at the national level to provide consistency and standardization of HT training content as well as to guide a process that would develop metrics for evaluation and the building of an evidence base.

**Abbreviations**: AAP: American Academy of Pediatrics; ACF: Administration for Children and Families; CME: Continuing medical education; ED: Emergency department; HCP: Health care professional; HEAL: Health, Education, Advocacy, and Linkage; HHS: United States Department of Health and Human Services; HT: Human trafficking; IOM: United States Institute of Medicine; MH: Mental health; NHTRC: National Human Trafficking Resource Center; SOAR: Stop, Observe, Ask, and Respond to Health and Wellness Training

## Introduction

Some 21 million adults and children are labor-trafficked or sex-trafficked through force, fraud, or coercion [1]. Visibility of this global phenomenon of human trafficking (HT) has reached the attention of health care professionals (HCPs) [2] as they provide care to victims of HT in hospital emergency departments [3–5], community health centers [6–8], migrant and refugee care centers, and adolescent health care centers [9]. In recognition of this interface between trafficking victims and the healthcare setting, over the last 10 years there has been a notable increase in training of HCPs on HT and its health implications. Many organizations – federal, state, local, non-governmental, academic, and professional societies – have developed curricula and offered training in various clinical settings [10]. However, methods and content of this education on trafficking vary widely, and there is little evaluation of the impact of this training.

### Current state of training on human trafficking

Training on human trafficking for HCPs has grown in parallel with the literature on the health effects of trafficking. A 2003 European study was the first to demonstrate the health risks and consequences of trafficking in women and adolescents [11]. In the years following that publication, more evidence has emerged about the health consequences of HT, the types of HCPs encountering trafficking victims, and the gaps in HCP knowledge about the problem [12–14]. In recognition of the importance of medical education on trafficking, medical professional societies and academics have called for HT awareness among family medicine practitioners [15], midwives [16], nurses [17,18], dentists [19], pediatricians [20], emergency department (ED) physicians [3–5] obstetricians-gynecologists [21], psychiatrists [22], and public health practitioners [23]. The American Academy of Pediatrics (AAP) has placed HT training in the top ten policies to be supported by its Board [24]. The AAP has further developed guidelines for pediatricians who may encounter victims of child sex trafficking and commercial sexual exploitation in their health care settings [9]. The American Academy of Family Physicians passed a resolution for HT awareness and education for practitioners of family medicine [25] as has the American College of Emergency Physicians [26]. Similar statements have been generated by the American Medical Association to encourage member groups and sections, as well as the Federation of State Medical Boards to raise awareness about HT and inform physicians about the resources available to aid them in identifying and serving victims of HT [27]. Moreover, the United States (US) Institute of Medicine (IOM), National Academy of Medicine, has released guidelines for confronting the commercial sexual exploitation and sex trafficking of minors [28]. Across the globe, academic medical centers and nonprofit organizations have undertaken initiatives to educate healthcare providers. For example, the International Organization for Migration, the National Human Trafficking Resource Center, the Massachusetts Medical Society, Children’s Healthcare of Atlanta, Mount Sinai Emergency Medical Department, American Medical Women’s Association, and Christian Medical and Dental Associations, have developed curricula on HT for HCPs [10]. In order to unify these national efforts, HEAL (Health, Education, Advocacy, and Linkage) Trafficking, a network of interdisciplinary professionals working on the intersection of public health and trafficking was founded in 2013. In addition to connecting the health experts working on HT, they have created an online compendium of medical literature and educational resources for HCPs on HT, and its Education and Training Group has worked closely with federal efforts on the topic [29].

### Federal action on training on human trafficking

In 2008, the Office of the Assistant Secretary for Planning and Evaluation (ASPE) in the US Department of Health and Human Services (HHS) acknowledged that there were challenges, barriers, and promising practices in addressing the needs of victims of HT [30]. In 2010, the ASPE released an issue brief, ‘Medical Treatment of Victims of Sexual Assault and Domestic Violence and Its Applicability to Victims of Human Trafficking’, which outlined several recommendations including the need for comprehensive screening practices, the importance of examination of protocols, and the content of effective training [31]. Since the 2008 ASPE National Symposium on the Health Needs of Human Trafficking Victims, HHS has committed to the Federal Strategic Action Plan (SAP) on Services for Victims of Human Trafficking in the United States (US) [32]. Released in 2013, the SAP was developed by the President’s Interagency Task Force to Monitor and Combat Trafficking in Persons and outlines the role of numerous federal agencies and offices working on the health response to trafficking including the Office for Victims of Crime, Office on Violence Against Women, the Federal Bureau of Investigation, Administration for Children and Families (ACF), Department of Homeland Security, Indian Health Services, and HHS. Furthermore, in 2014, the Office to Combat and Monitor Trafficking in Persons within the US Department of State issued a call to action for HCPs to combat HT [33]. In September 2013, ACF launched a pilot education initiative for HCPs, known as ‘Stop, Observe, Ask, and Respond to Health and Wellness Training (SOAR),’ and in June 2015, HHS established an Office on Trafficking in Persons within ACF.

The call for more training has led to recent proposals by advocates to the US Congress to support HT training for HCPs. The Trafficking Awareness Training for Health Care Act of 2015, initially proposed in 2014 (HR 5411) will complement HHS’s anti-trafficking efforts to engage the health care community by increasing information, awareness, and training for HCPs, not only in hospitals and community clinics, but in health professions schools, including schools of medicine, nursing, dentistry, and social work. The Trafficking Awareness Training for Health Care Act of 2015 was passed as a part of the Justice for Victims of Trafficking Act of 2015 [34]. Also, in 2015, S.1446, SOAR to Health and Wellness Act of 2015, was proposed, which would expand and further codify the SOAR training, diversify the reach of those facilities and individuals to be trained, and increase the types of training to be offered [35].

### Current status of evaluation of human trafficking training for healthcare providers

There have been few evaluation studies of HT training for HCPs. Most studies have conducted pre-testing and immediate post-testing, and a few examples are provided here. As part of a randomized control trial, emergency medical providers in major pediatric hospitals in the US in the San Francisco Bay area were trained in HT and then evaluated following that brief educational intervention. The results showed increased ED provider knowledge and self-reported recognition of HT victims [14]. In another study, using curriculum from *Caring for the Trafficked Persons*, a handbook developed by the International Organization for Migration (IOM) [36], training was conducted in seven countries in three regions (the Middle East, the Caribbean, and Central America) [37]. They identified training needs and misperceptions among HCPs about HT which would be useful in designing further programs for identification, care, and referral of HT victims. These studies looked at the training of HCPs in low- and middle-income countries, but not in high-resource countries. As part of its SOAR to Health and Wellness Training, HHS conducted a pilot of a series of HT trainings for 180 HCPs in six US cities in 2014 and then evaluated the training as a pre-test, post-test, and follow-up at three months, and participants across sites demonstrated a statistically significant increase in knowledge and attitude change on post-presentation evaluation [38]. Other recent studies have shown that HCP behavior changes as a result of education; those HCPs with training in HT were more likely to report HT, have encountered a victim in their practice, and have greater confidence in their ability to identify victims [20].

## Methodology

The goal of this study was to assess the gaps and strengths in HT education of HCP in the US. This mixed-method study had two components. The first component consisted of structured interviews with experts in human trafficking HCP education. The second portion of the study involved an analysis of data from HCP calls to the National Human Trafficking Resource Center (NHTRC).

### Interview analysis

For the expert interview portion, a convenience sample of 24 US-based experts was identified through snowball recruitment within the HEAL national network. All interviewees were actively engaged in HT education of HCPs for at least two years. All 24 individuals representing various US organizations and institutions were contacted initially by email explaining the nature of the study, how they were chosen, and inviting them to engage in a phone interview guided by a questionnaire on the topic of HT training for HCPs. All potential interviewees were instructed that their participation would be voluntary, not incentivized, and that information gathered would be anonymous. The interview captured trainer-specific data on types of HT training, duration and frequency, key content areas, presence of evaluation approaches and indicators as well as an assessment of barriers and strengths in HT training for HCP (Appendix 1). The interview questions were open-ended and the interview was conducted via telephone. Of the 24 invitees, 11 (46%) participated in phone interviews between May and June 2015. The non-responders among the original 24 had been contacted twice for participation; when no response was forthcoming, their names were excluded. The interviews lasted 40–70 minutes and detailed notes were taken during the interview. The data were analyzed for trends, and conclusions were drawn based on the composite sample. A content analysis to determine common themes was performed on the open-ended question portion of the expert interviews [39].

### NHTRC database analysis

Founded in 2007, and supported by grants from HHS, Polaris was charged with establishing and maintaining a NHTRC hotline for any caller in over 200 languages (interpretation via tele-translation services). The NHTRC provides information about anti-human trafficking resources to victims, concerned citizens, and service providers, including HCPs. In particular, a HCP may call the hotline for guidance about how to screen for trafficking as well as to find local resources for a potential trafficking victim. Call information captured typically includes the state in which the caller is located, the caller’s self-reported category or profession (e.g., medical professional, mental health professional, law enforcement, victim, or community member), the reason for calling (e.g., reporting a tip, referral, or general information), and the ‘awareness method’ of the caller (how they knew about the hotline, e.g., internet, prior knowledge, training, or word of mouth). Analysis of HCP calls to the NHTRC center provides an aggregate sense of the national trends in HCP awareness and behavior.

This project queried the Polaris Hotline database, through collaboration and support of Polaris staff, to determine various characteristics of the calls, such as overall trends in calls, including that for HCP and Mental Health (MH) providers, HCPs’ reasons for calling the hotline, and geographic trends. The data were shared in an aggregated, de-identified manner and represent numbers of signals in the form of phone calls, emails, and online tip reports received by the hotline. Of note, the timeframe for call analysis begins 1 January 2008 as this is the time period for comprehensive Polaris data collection. MH provider calls were first tracked starting in 2012, so the data for this subgroup of HCPs are only available from 2012 onwards.

The authors conducted three levels of data analysis on the NHTRC data (1) Total hotline annual calls from 2008–2014 were compared to those by HCPs (including MHs); statistical analysis using SAS © 64-bit 9.4 statistical software, was used to analyze differences in call volumes between total calls and HCP calls; (2) The nature of HCP calls over that same time period; and (3) Geographic trends in HCP calls, including calls in the three months following the HHS SOAR initiative pilot training.

For the statistical analysis in stage 1, we first assessed whether there was a change per year in the number of HCP calls and the overall number of calls. We used the regression model:

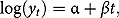

where y_t_ = number of calls in year t,and t = 0,1,…,7 for years 2008-2014.

The parameter 100% × [exp(*β*) – 1] can be interpreted as the rate of change per year in the number of calls.

To assess differences in rates of change between HCP calls and non-HCP calls over time, we created a dataset with 14 observations corresponding to each combination of year (2008–2014) and type of call (HCP/non-HCP). We then ran the regression model:

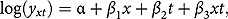

where

x = 1 for HCP calls, = 0 for non-HCP calls, t = years since 2008 = 0,1,…, 7, and

y_xt_ = number of calls of type x in year t.

The expression 100% x [exp(*β*_2_) – 1] can be interpreted as the rate of change per year in non-HCP calls.

The expression 100% x [exp(*β*_2+_ *β*_3_) – 1] can be interpreted as the rate of change per year in HCP calls.

Thus, to test whether the rate of change in HCP calls is the same as for non-HCP calls,we tested the hypothesis H_0_ : *β*_3_ ≠ 0.

## Results

Findings are categorized in two areas: results of interviews and results of the Polaris database analysis.

### Interview analysis

The results of the study were derived from interviews of eleven individuals currently conducting US-based training on HT for HCPs. Interviews revealed that the experience, approach, and content of such training varied widely. Two organizations had recognized the need for such training as early as 2002 and began that year, whereas others started HT courses as late as 2014. One quarter (27%) had been conducting HT training for HCPs since 2012. All training included core content, such as the global estimates of prevalence of HT, risk factors for HT, characteristics of victims and their traffickers, and basic identification of signs and symptoms of a possible HT victim within a health care setting. Case studies were commonly used to illustrate the key points. Most HT training material was developed in-house (91%), with some reliance on materials and resources developed by others.

Training venues included hospital-based medical grand rounds (55%), organizational offices (36%), as well as national medical and professional specialty conferences. The format was usually a live presentation, but in some instances training utilized on-site video sessions [40], on-demand internet based slide presentations, and live webinars. The number of participants at the training sessions ranged from 15–700. Most (73%) chose to provide on-site training in order to maximize interactions through such means as question-and-answer sessions, small group discussions, or presentations by HT survivors. Some training consisted of a single session with one presenter, as in a guest lecturer for medical grand rounds (55%); while other sessions were formulated as a panel of multidisciplinary speakers, including child abuse pediatricians, nurse practitioners, dentists, psychiatrists, psychologists, obstetricians, gynecologists, emergency medicine specialists, as well as licensed clinical social workers and PhDs with expertise in domestic violence / intimate partner violence. Some presentations (45%) included HT survivor presentations and stories in order to provide a first-hand perspective of the experience and impact of HT on an individual life; the others used case studies to illustrate those points.

The length of training sessions varied greatly. Availability of HCP time to set aside for training was one large barrier to delivering training. For example, one session type consisted of a focused, 20-minute presentation; whereas most were half-day (46%) or whole-day seminars and workshops. At one institution, HT training for HCPs was said not to be taken seriously until grant funding was awarded. The promotion by the AAP and the IOM Report [24,28] was noted to help increase awareness of the need for HT training for HCPs. Given local needs and increasing awareness about HT among HCPs, most presentations were repeated periodically over the years for different types of HCPs. About 82% had been conducted more than five times since inception. For those organizations and individuals who had been providing training over a few years, some degree of course adaption and adjustment occurred over the years, based on feedback from course participants as well as increasing experience in anti-HT activities by the trainers themselves. In some instances, the initial training focused on child abuse content and then over the years, it expanded to include commercial sexual exploitation of children and domestic violence or intimate partner violence. Many participants cited the need to conduct training of trainers as the demand for HT training for HCPs has increased and cannot be met by the original trainers or panelists; however, the challenge is to maintain quality control of presenters and presentations. Some degree of funding support for training was available; 63% (7/11) mentioned that they had received or had access to small grants to help cover costs of HT training; the others had in-kind support, such as access to a training site or panelists who volunteered their services.

Even though HT training for HCPs reported by our cohort was conducted as early as 2002, evaluation of training impact was generally lacking or underdeveloped. Pre-and post-tests were administered in only one-fifth (18%) of all presentation formats. These short tests were administered at the time of the training and captured immediate changes in knowledge and or attitude by HCPs about HT. In one instance there was a three-month follow-up; otherwise, most HT training had no formal impact measurement, although there were anecdotal reports of HT cases identified by a trainee, subsequent to training. Apart from such anecdotal reports, from our cohort and literature review, we identified that further studies are needed to demonstrate the long term HT training impact on HCP behavior and patient outcomes [20].

When queried about potential improvements in current training approaches, interviewees identified and commented upon the needs for: standardization of training, field-tested metrics for training impact in order to develop an evidence base, access to funding support, and incentivization of HCPs for training. Moreover, standardization of training material content was seen as important, especially when non-HCPs are organizing training on the health aspects of HT. The development of guidance, tools, and impact metrics (short-term and long-term targets and indicators) were mentioned as current needs. Some interviewees stated that successful applications for funding required quantitative evidence of training effectiveness. Lastly, they expressed the need for incentives for HCPs to initiate training. Two main incentivization categories were proposed: (1) continuing medical education (CME) credits awarded for participation in training; and (2) state requirements for HT training for professional licensure or re-licensure. All participants stated that HT training needed to move beyond knowledge to skill development and application.

Given these observations, the interviewees agreed that an authoritative national body should lead the charge and oversee the standardization of curriculum content, the development of robust metrics for impact evaluation, and a method to encourage broader participation by HCPs in HT training. Options discussed were the HHS ACF Office on Trafficking in Persons, with support by specialty societies and professional organizations, such as the American Medical Association, the American Academy of Pediatrics, the American Academy of Family Practitioners, the Society for Adolescent Medicine, the Christian Medical and Dental Associations, and the American Public Health Association. The researchers posit that human trafficking HCP curricular oversight could also be a role for HEAL Trafficking.

### NHTRC database analysis

**Figure 1.**
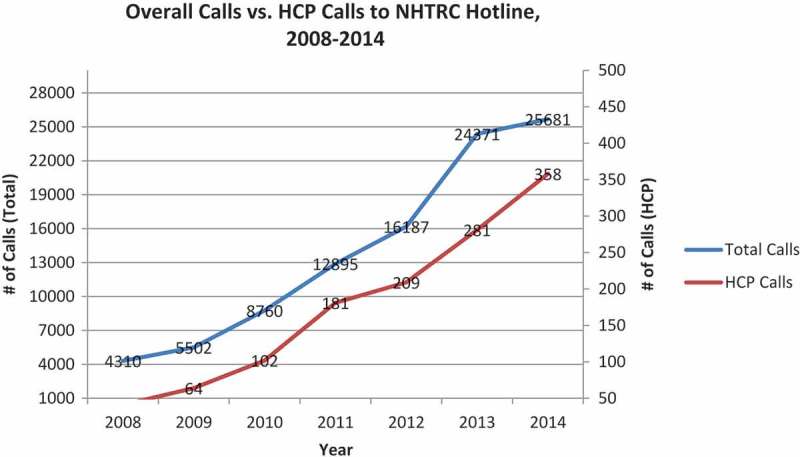
NHTRC calls- general vs. health care provider (HCP).

**Figure 2.**
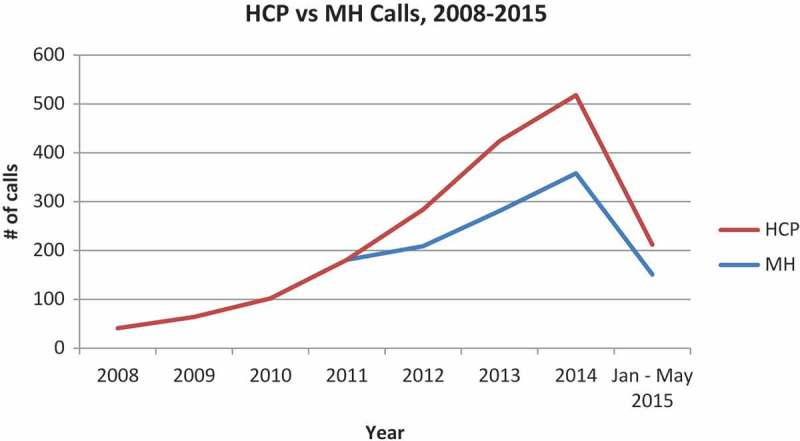
Total health care provider (HCP) calls vs. mental health (MH) provider calls to NHTRC.

Call data have been captured by Polaris through the NHTRC hotline since 1 January 2008. For the timeframe studied (1 January 2008 – 31 May 2015), 1826 calls were received from HCPs out of a total of 108 650 calls. The 1826 calls from HCPs included the 439 calls from mental health providers (MHs), which were reported separately beginning in 2012. Thus, overall HCPs represented about 1.7 % of all callers. Among the 40 caller types categorized by Polaris from 2008 to 2015, calls by HCPs have consistently ranked between 14th and 16th place, with MH ranking 24th or 25th. The total number of hotline calls increased steadily since inception of the database by NHTRC (Figure 1), and more general HCPs than MH providers have called over the years (Figure 2). The number of calls by medical professionals has always outnumbered those from mental health professionals, which may be a reflection of a lower number of mental health clinicians nationwide. Interestingly the percentage increase in HCPs calls from 2012–2014 was 71.29%, which is higher than the overall percentage increase of calls to the hotline over that same time period of 58.65%. Overall, the there is a statistically significant rise in both general and HCP calls, by year, from 2018–2014 (p < 0.001), and while HCP calls show a greater increase than general calls, the rate of increase is not statistically significant in comparison to general calls.

**Figure 3.**
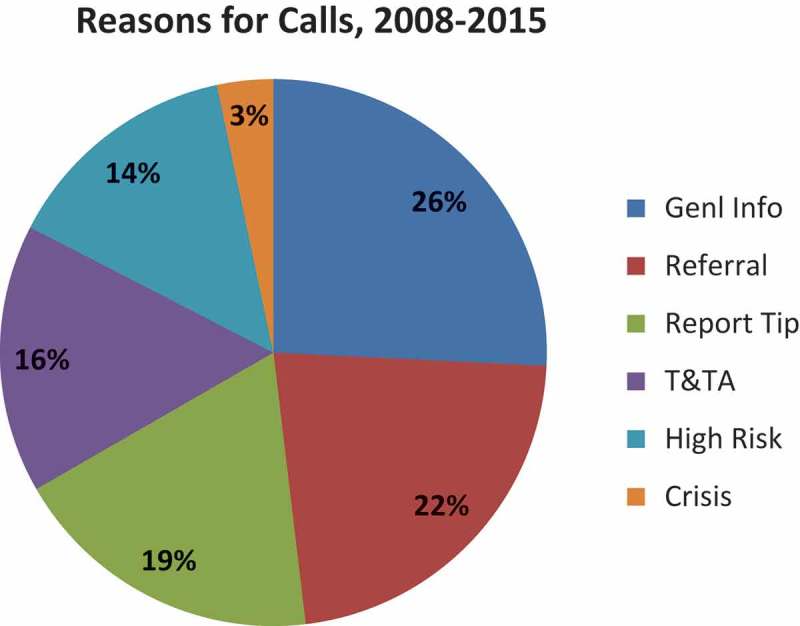
Reasons for health care provider (HCP) calls to NHTRC. The NHTRC utilizes seven distinct categories to describe a caller’s reason for contacting the NHTRC and track substantive calls received through the hotline. Substantive calls exclude hang ups, wrong numbers, missed calls, and calls where the caller hangs up or is disconnected before the purpose of the call can be determined. General Information Requests: This category includes calls requesting general information on the issue of human trafficking, such as legal definitions, scope, statistics, trends, and prevalence. Direct Services Referral Requests: This call category includes requests for direct service referrals for survivors of human trafficking. Referrals may include contact information for service providers, law enforcement, coalitions and other collaborative efforts, and other relevant agencies or field practitioners. The most commonly requested referrals are for case management services, shelter services, legal services, mental health or medical services. Tips: This category includes calls received from individuals who wish to report tips related to human trafficking victims, suspicious behaviors, and/or locations where human trafficking is suspected to occur. Potential human trafficking tips received by the NHTRC are reviewed by hotline supervisors and regional specialists before being passed on to the appropriate local, state, or federal investigative and/or social service agency equipped to investigate and/or respond to the needs of victims. Not all tips are reported to law enforcement, and any reports made respect callers’ preferences regarding confideniality. Reporting decisions are based on a variety of factors, including the callers’ needs and wishes, and the needs and wishes of victims. Training & Technical Assistance (T&TA): T&TA requests include but are not limited to: specialized information; programmatic and project support; phone consultations; materials reviews; and trainings and presentations. At-Risk: This category refers to calls referencing related forms of abuse and exploitation that may put individuals or specific populations at risk for human trafficking, such as labor exploitation, domestic violence, sexual assault, child abuse, and runaway/homeless youth. Crisis Calls: This category includes calls received from victims of human trafficking in need of immediate assistance or from an individual calling on behalf of a victim in need of immediate assistance or emergency services. The NHTRC has developed extensive crisis protocols and local emergency referral and reporting networks to ensure that NHTRC staff are able to provide an immediate and tailored response to crisis calls. Unrelated: This call category refers to calls that are outside the scope of NHTRC services. NHTRC Call Specialists refer callers to other national hotlines, service providers, or coalitions that are best equipped to fulfill their request.

**Figure 4.**
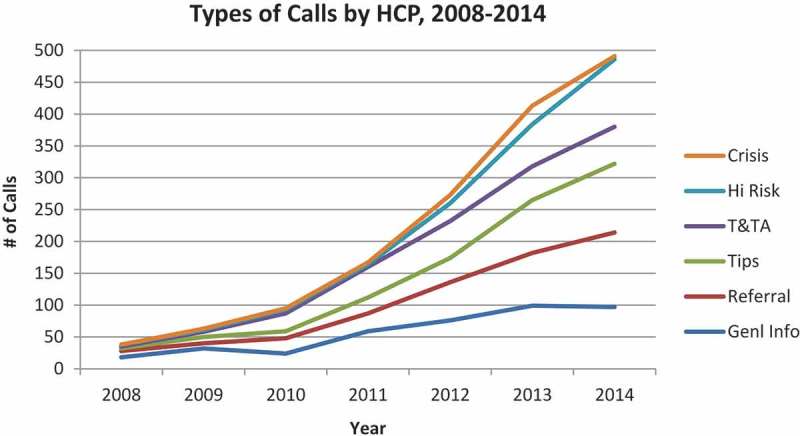
Types of calls by health care provider (HCP) to NHTRC over time.

The types of NHTRC calls for HCPs are categorized as: general information requests; direct services referral requests; tip reports; training and technical assistance (T and TA); at-risk; crisis; and unrelated. For definitions of these categories, see caption to Figure 3. Regarding reasons for calling, approximately one-half of all calls by HCPs (medical and mental health professionals) were for general information or referrals (Figure 3). Trends for these data by category of call by HCP show a doubling in 2013 in calls about high risk cases and a peak in crisis assistance calls in 2012 and 2013 (Figure 4).

Geographically, the dataset indicates calls by caller location at the state level and combines calls from HCPs and MHs. The reader can refer to the NHTRC website for comparisons of total calls by state as far back as 2012: http://traffickingresourcecenter.org/states. Polaris has received calls from HCPs in 48 states and Washington, DC, but no calls from HCP in South Dakota or Wyoming. California and Texas have always led the list of states from which HCP calls have been made, with Ohio and Florida HCPs showing up as frequent callers in the top five listing (Table 1). These numbers may reflect the larger absolute number of HCPs and larger general populations.

**Table 1.** NHTRC number of HCP calls by state, 2008–2015.

State	Number of Calls, 2008–2015
California	279
Texas	173
Florida	99
Ohio	98
Illinois	71
New York	67
Washington (state)	61
North Carolina	58
Pennsylvania	57
Massachusetts	56
Michigan	55
Maryland	54
Georgia	53
Missouri	43
Virginia	42

Lastly, we examined the quantity of HCP calls from states in which the federal SOAR pilot training was conducted. We found that there were fewer calls per month in the three months right after the training, compared to the eight months before the training. However, there may be multiple explanations for this, including a greater awareness of local resources following training, and a decreased need for national technical assistance.

## Discussion

### Main findings

Analysis of interviews with human trafficking HCP educators revealed a breadth of training modalities and content, which mirrors a recently published literature review on the topic [10]. The process of curricula development was described as organic. Training had similar content areas, including global estimates of prevalence of HT, risk factors for HT, characteristics of victims and their traffickers, basic identification of signs and symptoms of a possible HT victim within a health care setting, and suggested initial response by the HCP. When queried about potential improvements in current training approaches, standardization of training, metrics to evaluate and develop the evidence base for training impact, funding opportunities, survivor integration, and incentives to encourage training were common themes.

Analysis of the 2008–2014 HCP data from the National Human Trafficking Resource Center (NHTRC) is one way of capturing national behavior change among HCPs. In other words, by calling the NHTRC Polaris hotline, a HCP is engaging in an activity that comes as a result of awareness of the problem of trafficking. While HCPs may contact local service organizations directly, the NHTRC call specialists are analogous to poison control centers, in that they provide technical expertise to guide a clinician through a specialized clinical case, human trafficking. Call specialists can outline case-by-case victim resources, provide guidance for screening questions to ask patients, and can speak to the patient directly in over 200 languages (via tele-interpretive services). While HCP calls have paralleled the general national hotline trend data, over the last couple years, the relative increases in HCP calls have surpassed that of the general population. These trends indicate that HCPs are increasingly aware of trafficking and may indicate some level of behavior change.

### Recommendations

**Figure 5.**
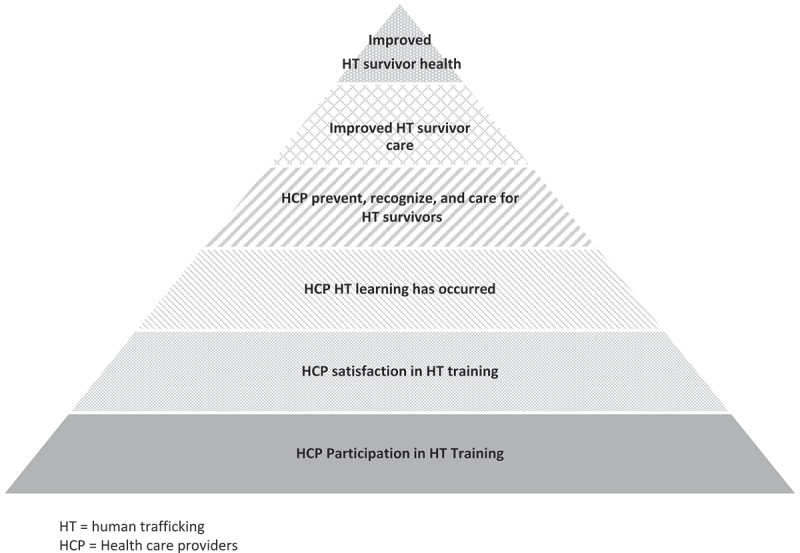
Assessing impact of human trafficking medical education, a hierarchical, patient-centered model. HT = human trafficking; HCP = Health care providers.

- **Content and delivery**: Standardize content of training so that key information is correctly and consistently provided to all HCPs and training participants, regardless of venue, format, or level of experience among HCPs. Content should include primary, secondary and tertiary HT prevention, as well as public health impact using the socioecological model grounded in an understanding of links to other forms of intentional violence including intimate partner violence, child abuse, and community violence. The survivor voice should be included to provide first-hand perspective [41]. Moreover, all forms of trafficking should be covered, including labor, sex, and organ trafficking. The training should be victim-centered, culturally-relevant, evidence-based, gender-sensitive, and trauma-informed. Content delivery should not be limited to formal didactic presentations, as medical education literature [42] has shown that more interactive and innovative strategies, such as simulated patients, flipped-classrooms, faculty modeling, and role-playing, are often more effective [43].
- **Evaluation and metrics** Develop evaluation metrics specific to HT training for HCPs such that changes in knowledge, attitude, and practice, as well as patient outcomes can be reliably and reproducibly measured, as well as compared across training types for generalizability [44]. This step will help to build the evidence base for effectiveness and impact of HT training for HCPs and allow adaptation and improvement when promising practices are identified. This measurement should go beyond knowledge acquisition, trainee behavior change, and process analysis to long-term impact analysis on trafficked patients – namely, patient-centered outcomes [45,46]. See adapted Kirkpatrick model (Figure 5).
- **Oversight**: For standardization and evaluation, the optimum choice of agency or institution to provide US-based oversight should be one that has a national scope and authority, a specific interest and expertise in HT training for HCPs, and no conflict of interest. A federal agency such as HHS ACF would be well positioned to provide such oversight and guidance. They could work in conjunction with professional specialty societies (e.g., AAP, AMA, Society for Adolescent Medicine, APHA, CMDA) and HEAL Trafficking.
- **Research**: Conduct and publish research using rigorous study designs, methodologies, and outcome measures that demonstrate practices that lead to provider practice change and improved patient outcomes [47]. A national oversight committee or commission could facilitate the dissemination of the findings and convene periodic forums to discuss the implications and next steps.
- **Advocacy**: Research and identify the best means to incentivize HCPs to train in HT. A legislative approach may not be ideal, as it could lead to an unfunded mandate. A group such as the Federation of State Medical Boards could explore the opportunities and challenges to linking state licensure and re-licensure to HT training. Thus far, it is worthwhile to note that a growing number of states are mandating HCP HT education, including Michigan [48] and New Jersey [49]. Other entities could explore the incorporation of HT training into health professional school curricula, and residency training, as well as the best approach for offering CME.
- **Collaboration**: Develop a ‘Quality Improvement Collaborative’ (QIC) – a network of groups which conduct training and which would be willing to coordinate an evidence base regarding the above mentioned recommendations. The development of QICs could allow for a gathering of invested individuals to learn, reflect, and share strategies [50], ultimately towards the end of enhanced research, innovation, advocacy, and funding around topics of HT training for HCPs.
- **Funding**: Use data and published research to advocate for funding for initiatives around HT training for multidisciplinary HCPs. This funding should not take away resources from existing service provision for survivors.

### Limitations

This paper used a mixed methods approach to understand the overall state of medical education for HCP on HT. Our HT HCP educator interview analysis has the richness of a breadth of perspectives, however due to snowball recruitment, is not generalizable. There are many ongoing training sessions on HT for HCPs, and it was not feasible to list all those and then sample from that list. Our analysis of National Human Trafficking Resource dataset was limited by its aggregate form, so we were only able to present general trends.

## Conclusions

Human trafficking is a health issue, and as such HCPs have the potential to play a critical role in human trafficking victim prevention, identification, and care [51]. While some HT training for HCPs began over 10 years ago, most HCP are unfamiliar with how to care for trafficking survivors. The Transtheoretical Model states that awareness and knowledge precede behavior change. Furthermore, behavior change comes in stages, from pre-contemplation to action [45,52]. The pinnacle of successful medical education is improved patient outcomes [45]. Our review of National Human Trafficking Resource Center Data confirms the growing awareness of trafficking among HCPs, and possibly indicates some level of behavior change. Our interviews with HT educators demonstrate that the standardization and evidence on medical education of HT has many opportunities for growth. The field is wide open for progress to be made by scholars, clinicians, and other practitioners. Given that victims of HT will interact with a variety of clinicians throughout their care, HT training should include the whole range of HCPs. Standardization is essential to ensure that content is correct, consistent, relevant to HCPs, and effective in practice. Training should incorporate an evidence-based, patient-centered, trauma-informed approach, with proven effectiveness. Moreover, training should incorporate orientation on gender, cultural competencies, and survivor input. Steps need to be taken to explore how such training might be integrated into settings such as health professional school curricula, residency programs, and possibly to determine whether HT training could be a requirement for licensure or re-licensure of HCPs. Identification of HT by HCPs and treatment protocols will need to be researched and funded in order to expand that evidence base [53,54]. Post training evaluation must go beyond the immediate measurement of changes in knowledge and attitudes to assess for patient-centered outcomes. Metrics with indicators and targets need to be developed, field tested, and disseminated for general application where HT training is conducted. Training effectiveness measured by changed HCP behavior, along with improved patient outcomes, i.e. earlier identification of and support for HT victims, are the ultimate goals. The larger initiative is best served by the establishment of a national oversight body which has both authority and convening power. That body can guide these processes, convene forums for identifying promising practices, assist QI collaboratives, and facilitate linkages with states, academia, and professional societies, to improve HCP engagement with trafficked persons presenting in clinical settings.

## Appendix 1

**Table:**

Interview Questions
**Respondent Information**
Location of Organization
Date of Interview
**Training Logistics and Format**
Where have you conducted training?
Do you offer more than one level or kind of training? If so, what kind?
In what year was the first training session you conducted?
What type of recruitment/advertisement of training was used?
How many training sessions have you conducted or offered?
Who were the populations in attendance at your training sessions?
Roughly how many people attend or participate (if online) at an average training?
Did you develop your own training materials, or did you use a training that was already developed?
If you used other off-the-shelf materials, whose did you use?
What was the format for training?
How long were the training sessions?
Which of the following components are covered in your training?
**Evaluation**
Was there formal post-training evaluation?
How did you conduct the feedback evaluation?
If there was formal post-training evaluation, how soon after the training session was this done?
If an evaluation was conducted, what was the general feedback received? *
Feedback in mind, if you were to administer the training again what would you do the same?*
What would you do differently? *
What has been the impact of the training? What examples of behavior change, practice changes, etc., are you aware of? *
**Next Steps**
What are the biggest barriers in training healthcare providers on human trafficking?
What do you perceive to be your next steps with regard to training HCP on HT?

All questions multiple choice, except those marked as otherwise

* Open-Ended
